# Diffuse parenchymal pulmonary amyloidosis associated with multiple myeloma: a case report and systematic review of the literature

**DOI:** 10.1186/s12885-018-4565-5

**Published:** 2018-08-08

**Authors:** Yin Liu, Zhibin Jin, Haiyan Zhang, Yingwei Zhang, Minke Shi, Fanqing Meng, Qi Sun, Hourong Cai

**Affiliations:** 1 Department of Respiratory, Nanjing Drum Tower Hospital, Nanjing University Medical School, 321 Zhongshan Road, Nanjing, 210008 Jiangsu China; 2 Department of Ultrasound, Nanjing Drum Tower Hospital, Nanjing University Medical School, 321 Zhongshan Road, Nanjing, 210008 Jiangsu China; 3 Department of Respiratory, Huainan Chaoyang Hospital, 15 Renmin South Road, Huainan, 232000 Anhui China; 4 Department of Thoracic Surgery, Nanjing Drum Tower Hospital, Nanjing University Medical School, 321 Zhongshan Road, Nanjing, 210008 Jiangsu China; 5 Department of Pathology, Nanjing Drum Tower Hospital, Nanjing University Medical School, 321 Zhongshan Road, Nanjing, 210008 Jiangsu China

**Keywords:** Diffuse parenchymal pulmonary amyloidosis, Multiple myeloma, Amyloidosis

## Abstract

**Background:**

Pulmonary is an uncommon site of extramedullary involvement in multiple myeloma (MM). Diffuse parenchymal amyloidosis as pulmonary manifestation of MM is even rarer. We report a rare case of diffuse parenchymal pulmonary amyloidosis associated with MM diagnosed by video-assisted thoracoscopic lung biopsy (VATLB).

**Case presentation:**

A 58-year-old woman complained of cough and shortness of breath. HRCT disclosed diffuse ground-glass opacifications with interlobular septal thickening in bilateral lungs. A lung-biopsy sample obtained by VATLB revealed Congo Red-positive amorphous eosinophilic deposits in the alveolar septa. Surgical biopsy of abdominal wall skin and subcutaneous fat was also performed, which showed the apple-green birefringence with polarized light on Congo red stain was demonstrated in dermis. The serum immunoelectrophoresis showed monoclonal lambda light chains. A bone marrow biopsy specimen comprised 11.5% plasma cells. She was therefore diagnosed with diffuse parenchymal pulmonary amyloidosis accompanied by MM. The patient was referred to the hematology department for further chemotherapy.

**Conclusions:**

It is important to recognize diffuse parenchymal pulmonary amyloidosis to avoid misdiagnosis.

## Background

Amyloidosis is a rare disease characterized by the deposition of insoluble misfolded proteins in various tissues and organs. Approximately 6–10 cases occur annually per 100,000 in western Europe and the United States [1–3]. But the exact incidence is unknown.

The respiratory system is involved in 50% of patients with amyloidosis, although radiographic demonstration is much less common [4, 5]. The respiratory amyloidosis may be localized or part of systemic amyloidosis. The three main types of respiratory involvement are tracheobronchial, nodular parenchymal, and diffuse parenchymal pulmonary amyloidosis [4].

Diffuse parenchymal pulmonary amyloidosis, also known as diffuse alveolar-septal amyloidosis, is the least common type of pulmonary amyloidosis. This type is sometimes seen in patients with multiple myeloma (MM) and is associated with a poor prognosis [6]. In this article, we will describe the clinical characteristics of a patient with diffuse parenchymal pulmonary amyloidosis associated with MM diagnosed by video-assisted thoracoscopic lung biopsy (VATLB) in our hospital to improve our understanding of this disease.

## Case presentation

A 58-year-old woman presented with a 2-year history of a non-productive cough and progressive shortness of breath. She had a history of renal insufficiency and persistent proteinuria, without any extra-renal involvement. She was diagnosed with IgA nephropathy for 15 years and had received immunosuppressive therapy for 6 years.

Her vital signs were stable at initial examination; the patient was afebrile and oxygen saturation was 95% in ambient air. On physical examination, auscultation of the lungs detected slight coarse crackles at the bilateral bases. The remainder of the examination was unremarkable.

**Fig. 1:**
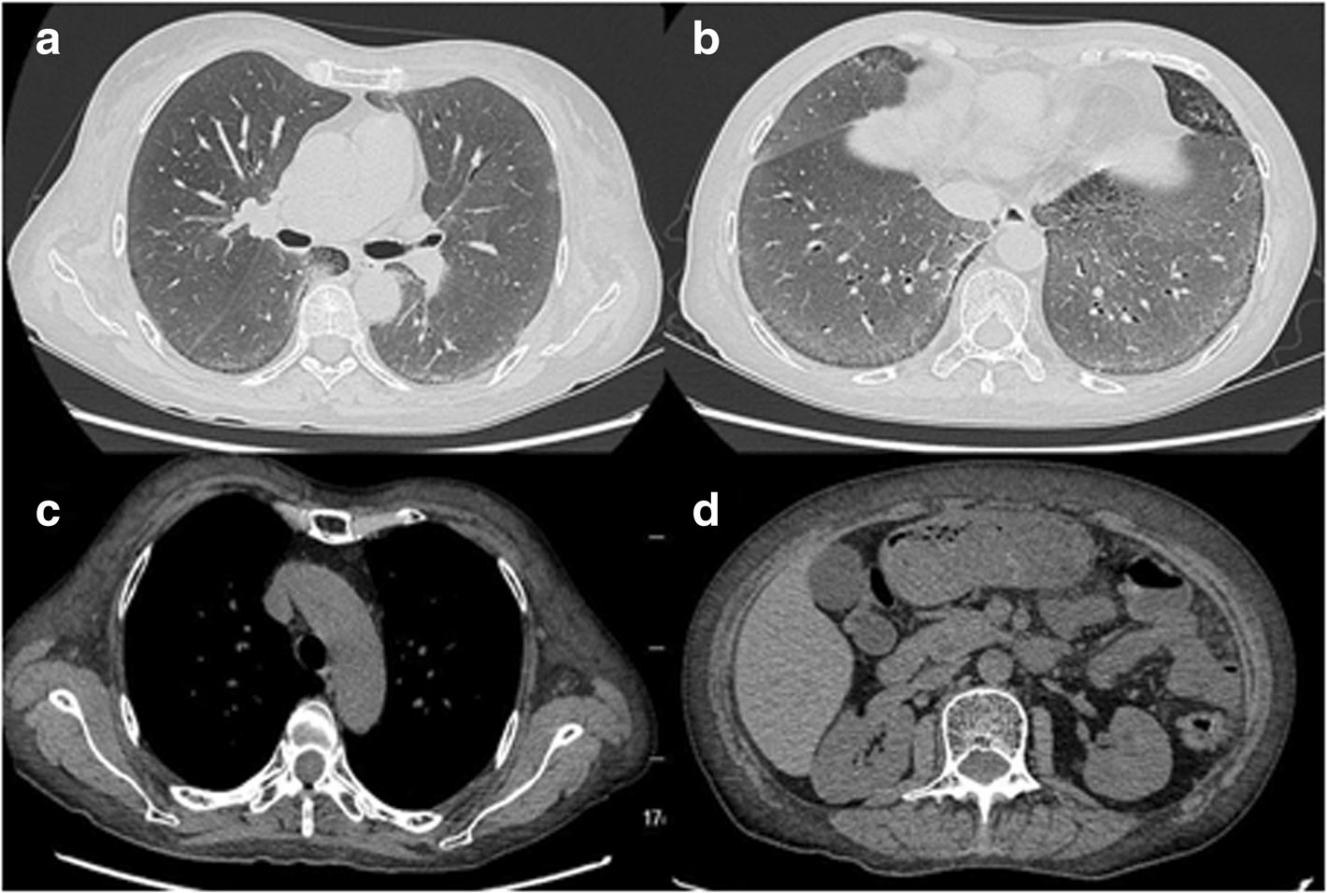
Chest and abdominal CT. (**a**, **b**) showed ground-glass opacities with interlobular septal thickening in bilateral lungs. (**c**, **d**) showed soft tissue infiltration of the subcutaneous fat layer with asymmetric bulging of the chest and abdominal wall

**Fig. 2:**
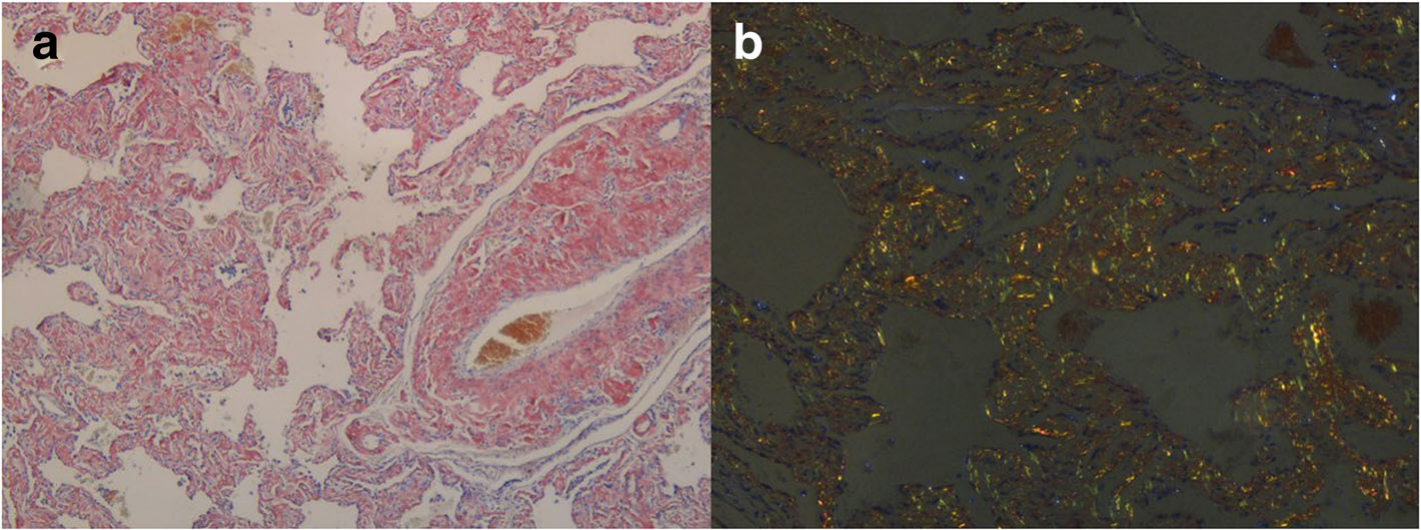
Histopathology of lung biopsy. **a** Congo red staining showed marked thickening of the alveolar wall deposition of amorphous eosinophilic amyloid at the bronchial mucosa, pulmonary vessel wall and interstitium. **b** Congo red staining display apple-green birefringence in polarised light

**Fig. 3:**
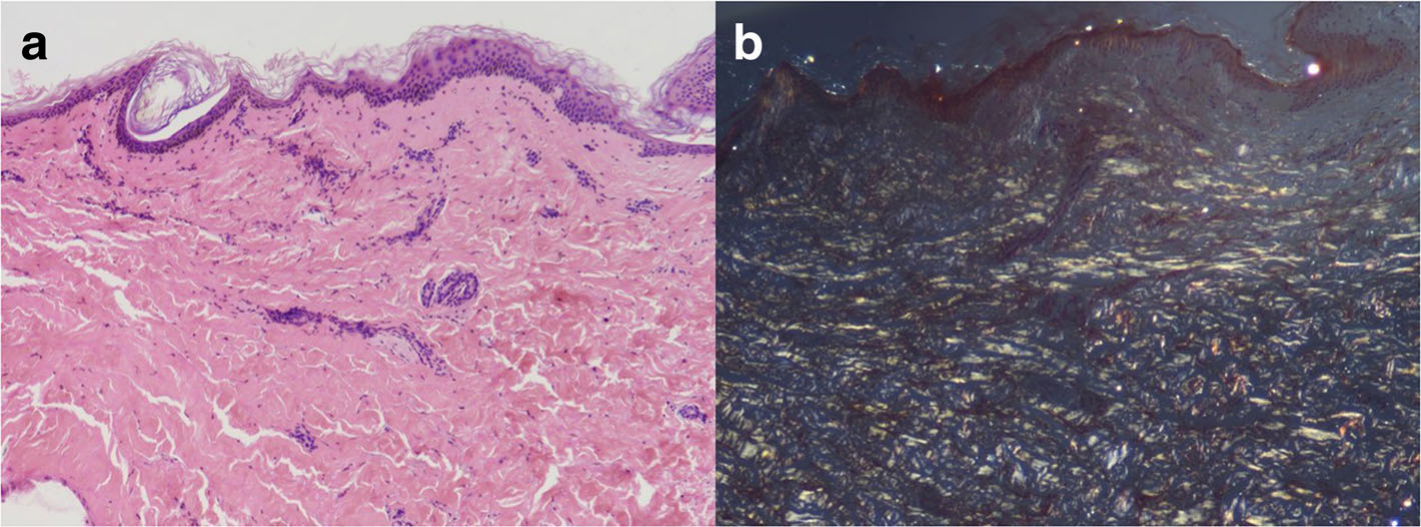
Histopathology of abdominal wall skin and subcutaneous fat. **a** Hematoxylin and eosin (H&E) staining of the biopsy specimen revealed deposits of acellular amyloid matrix in dermis. **b** The apple-green birefringence with polarized light on Congo red stain was demonstrated in dermis

Her hemoglobin was 95 g/L but renal function and calcium level were normal. The patient was negative for antinuclear and antineutrophil cytoplasmic antibodies in immunofluorescence assays. Repeated exams of sputum smear did not yield any pathogenic micro-organisms. Serum free light chain analysis showed measuring lambda light chain of 2.59 g/L. Serum protein electrophoresis revealed low IgG, IgA, and IgM levels, with reported immunoelectrophoresis (IEP) showing monoclonal lambda light chain peak with the monoclonal protein. A 24-h urine contained 5521 mg of protein. High-resolution chest CT revealed disclosed ground-glass opacities (GGOs) with interlobular septal thickening in bilateral lungs (Fig. 1a and b). Chest and abdominal CT shows soft tissue infiltration of the subcutaneous fat layer with asymmetric bulging of the chest and abdominal wall (Fig. 1c and d). Pulmonary function tests revealed a moderately restrictive ventilation disorder, with a forced vital capacity (FVC) of 1.76 L (69.0% of predicted) and severe reduction of diffusion capacity (DLCO SB 20.8% of predicted). Cardiac biomarkers, such as natriuretic peptides, particularly B-type natriuretic peptide (BNP) and cardiac troponin-T were normal. Echocardiogram showed normal left ventricular ejection fraction of 61% and there were no features of cardiac amyloidosis. A VATLB was performed, which showed marked thickening of the alveolar wall deposition of amorphous eosinophilic amyloid at the bronchial mucosa, pulmonary vessel wall and interstitium. Congo red staining display apple-green birefringence in polarised light (Fig. 2a and b). The immunohistochemical stain for protein A was negative for secondary amyloid. Following the VATLB results, bone marrow examination was performed. Bone marrow examination showed 11.5% plasma cells with lambda light chain restriction. Bone marrow cells by flow cytometry exhibited a typical phenotype for plasma cells, expressing monoclonal cytoplasmatic CD138, CD38, and cLambda instead of CD19, CD56 and cKappa, which are characteristic of the typical immunophenotype of MM. Fluorescence in situ hybridization (FISH) was carried out to check on the bone marrow aspirate. The most frequent abnormality in the patients was 1q21 amplification (25%), followed by 14q32 (IGH) translocations (29.5%), without 13q14 (RB1) deletion, 13q14.3 (D13S319) deletion and p53 deletion. No lytic bone lesions were detected with positron emission tomography/computed tomography (PET/CT). This confirmed the diagnosis of MM, λ light chain type, stage I. Surgical biopsy of abdominal wall skin and subcutaneous fat was also performed, which showed the apple-green birefringence with polarized light on Congo red stain was demonstrated in dermis (Fig. 3a and b).

She was therefore diagnosed with diffuse parenchymal pulmonary amyloidosis accompanied by MM. The patient was referred to the hematology department, where the patient started chemotherapy with bortezomib and dexamethasone, and her symptoms relieved 2 months after the initial presentation. No adverse effects were observed and the laboratory results were stable.

## Discussion and conclusion

MM accounts for 1% of all cancers and 10% of all hematologic malignancies [7]. Amyloidosis is a rare complication associated with MM. The most commonly affected organs are the kidneys, heart, spleen, lymph nodes and the liver [8]. Pulmonary is an uncommon site of extramedullary involvement in MM. Diffuse parenchymal amyloidosis as pulmonary manifestation of MM is even rarer; only a few cases have been reported [8–14].

Diffuse parenchymal amyloidosis is characterized by the presence of amyloid deposits in the alveolar septa and vessel walls. Pathologic examination of diffuse parenchymal pulmonary amyloidosis shows deposition of amorphous eosinophilic amyloid in the alveolar septa, especially around the capillary vessels [15]. Therefore, HRCT findings in such patients mainly comprise GGOs, interlobular septal thickening, intralobular reticular opacity, and nodules [1, 16]. Diffuse amyloidosis is sometimes accompanied by mediastinal lymphadenopathy. Pleural effusion may be present and occasionally dominate the clinical course. Multiple cysts and calcification probably resulting from fragile alveolar walls as a consequence of amyloid deposition both on alveolar walls and around capillaries have been described [17]. Differential considerations of diffuse parenchymal pulmonary amyloidosis are quite broad and include pneumonia, pneumoconiosis, interstitial lung disease and lymphangitic carcinomatosis.

Diffuse parenchymal pulmonary amyloidosis has a remarkably different, more clinical presentation. Such patients may develop symptoms of coughing and shortness of breath secondary to the amyloid deposits. It is characterized by widespread amyloid deposition involving small vessels and the interstitium [18]. This is reflected by lung function tests showing a restrictive pattern with reduced diffusion capacity of carbon monoxide and hypoxaemia upon exertion [8]. Affected individuals are more likely to progress to pulmonary hypertension and respiratory failure [1].

Tissue biopsy is the gold standard for the diagnosis and typing of amyloidosis. Diagnosis of amyloidosis is confirmed by the presence of apple-green birefringence under polarized light of a tissue biopsy stained with Congo Red [5]. The diagnosis of pulmonary amyloidosis is extremely important and requires histological analysis to differentiate it from other interstitial lung diseases. The diagnosis of pulmonary amyloid can be made by fiberoptic bronchoscopy, VATLB, and open thoracotomy. Although transbronchial lung biopsy (TBLB) is useful in some amyloidosis cases, it may be of less efficacy in establishing diagnosis due to the limit of the amount biopsy sampling. The lung VATLB is highly specific and sensitive. The biopsy of a clinically suspected organ is an invasive procedure and may be associated with complications including hemorrhage. Because amyloidosis is a systemic disease, routine biopsies from nonsymptomatic sites including the rectal mucosa, abdominal fat pad and labial salivary glands are more commonly used [19]. But, clinical and radiological manifestations of subcutaneous amyloidosis are very rare. In our case, diffuse soft tissue infiltration of the subcutaneous fat layer with asymmetric bulging of the chest and abdominal wall were demonstrated on CT. Surgical skin biopsy including the subcutaneous fat pad can be performed safely and is useful for diagnosing amyloidosis [20, 21].

Once the diagnosis is clear, diffuse parenchymal pulmonary amyloidosis requires intervention. The therapeutic goal for patients with concurrent pulmonary amyloidosis and MM is suppression of the production of amyloid protein comprising immunoglobulin light chain [7, 22]. Monoclonal antibodies such as daratumumab (Dara, human IgG1 anti-CD38) have shown promising efficacy for the treatment of relapsed and refractory MM and heavily pretreated amyloidosis [23, 24]. Therapy was well tolerated. Prospective studies of daratumumab alone or in combination with chemotherapy are warranted. Treatment of systemic amyloidosis aims at reducing the clonal cell populations producing amyloidogenic immunoglobulins, using high-dose chemotherapy followed by autologous stem cell transplantation in carefully selected patients. Its efficiency in treating diffuse pulmonary amyloidosis has not been established. Lung transplantation for isolated pulmonary amyloidosis has been reported [25]. The AL amyloid then contributed to pulmonary hypertension (PH) with severe symptoms necessitating lung transplantation. Ellender et al. described a case with PH from amyloidosis secondary to systemic lupus erythematosus and Sjögren’s syndrome, the patient received bilateral lung transplantation and remained stable after 7 years post lung transplantation [26]. Lung transplantation for isolated pulmonary amyloidosis or combined with PH may be performed in highly selected patients with good long-term outcome.

Diffuse parenchymal amyloidosis is usually a systemic phenomenon with a poor prognosis. A median survival for untreated patients is 13 months, and with the development of heart failure the survival duration decrease to less than 4 months [18]. When compared with nodular pulmonary amyloidosis, patients with diffuse parenchymal pulmonary amyloidosis has a far worse prognosis [10]. Gradual worsening of pulmonary function and symptoms is typical.

In conclusion, diffuse parenchymal pulmonary amyloidosis is a fatal disorder that is rare and often undiagnosed. Radiologists and physicians should consider amyloid in clinically perplexing chronically ill patients, particularly those with plasma cell dyscrasias or chronic inflammatory states. Tissue biopsy is the gold standard. With the use of subcutaneous fat pad and lung biopsy, an early diagnosis can be made.

## Abbreviations

BNP: B-type natriuretic peptide
FISH: Fluorescence in situ hybridization
FVC: Forced vital capacity
GGOs: Ground-glass opacities
IEP: Immunoelectrophoresis
MM: Multiple myeloma
PET/CT: Positron emission tomography/computed tomography
PH: Pulmonary hypertension
TBLB: Transbronchial lung biopsy
VATLB: Video-assisted thoracoscopic lung biopsy
